# Supplementary material for: The Spectrum of Cognitive Dysfunction in Amyotrophic Lateral Sclerosis: An Update
Source: Int J Mol Sci. 2023 Sep 27;24(19):14647. doi: 10.3390/ijms241914647 (PMC10572320; doi:10.3390/ijms241914647)
Supplement: Supplementary file 1 [file ijms-24-14647-s001.zip › ijms-2573092-supplementary.pdf]

Table S1. Brain network dysfunctions in ALSci.

Involved network	References
Limbo-thalamo-cortical pathway	[106]
Fronto-striatal network	[103]
Motor and cognitive network	[122]
DMN connectivity	[120,121]
Inferior frontal-occipital connection;<br>corpus callosum, uncinate fasciculi	[98]
Bilateral corticospinal tract & corpus callosum	[96]
Superior longitudinal fasciculi and corpus callosum / reduced FC in sensorimotor cortex-resting state network, frontal pole, bilateral thalamus-visual cortex network, increased FC in left primary motor cortex and left fronto-parietal network	[85]
Superior longitudinal & inferior fronto-occipital fasciculi	[93]
Frontotemporal & hippocampal white matter tracts	[96]
Frontal functional network	[124]
Frontal and global network	[122]
Multimodal cortico-basal ganglia network	[128]
Fronto-striatal and fronto-temporal circuit	[133]
Fronto-striatal and fronto-parietal circuit	[137]
Basal ganglia - frontal lobe & limbic circuits	[129,131]
Medial cingulate gyrus - prefrontal circuit	[134]
Bilateral motor and fronto-temporal cortex	[138]
Reduced FC: bilateral hippocampus, parahippocampal gyrus, posterior cingulate gyrus (Papez circuit)	[99]
Increased FC: sensorimotor, default mode, bilateral fronto-parietal and orbitofrontal networks	[129]
Increased FC: limbic, DMN, salient/ventral attention + fronto-parietal networks<br>Decreased FC: limbic and subcortical network	[130]
Corpus callosum, cingulum, inferior longitudinal, inferior fronto-occipital and uncinate fasciculus	[139]
Fronto-parietal and salience networks, DMN and SMN	[141]
DMN-somatomotor-frontoparietal and salience network	[142]
Fronto-tempo-parietal-cerebellar circuits	[221]
Reduced FC:<br>Sensorimotor, DMV, frontoparietal & orbitofrontal<br>Frontoparietal & sensorimotor network<br>Frontoparietal, frontotemporal & resting state network	 [129]<br>[222]<br>[138]
Increased FC (initial):<br>Middel & superior frontal, frontoparietal, frontostriatal network<br>Decreased FC (later):<br>Between medial frontal gyrus & parietal regions	[137]

ALSci: amyotrophic lateral sclerosis with cognitive impairment; FC: functional connectivity; DMN: default mode network; SMN: sensorimotor network
